# Accuracy of the preoperative diagnostic workup in patients with head and neck cancers undergoing neck dissection in terms of nodal metastases

**DOI:** 10.1007/s00405-020-06324-w

**Published:** 2020-08-29

**Authors:** Angéla Horváth, Péter Prekopp, Gábor Polony, Eszter Székely, László Tamás, Kornél Dános

**Affiliations:** 1 Department of Otorhinolaryngology, Head and Neck Surgery, Semmelweis University, Szigony Str. 36, Budapest, 1083 Hungary; 2 2nd Department of Pathology, Semmelweis University, Budapest, Hungary

**Keywords:** Imaging, Head and neck, Squamous cell carcinoma, Ultrasound, FNAC, Neck dissection

## Abstract

**Purpose:**

The presence of cervical lymph node metastases is one of the most influential prognostic factors in head and neck squamous cell carcinomas. The management of clinically N0 neck in patients with head and neck cancer remains controversial: elective neck dissection has relatively high morbidity, adversely affecting the quality of life, however, abandoning elective neck dissection is known to compromise overall survival in numerous primaries. The purpose of this study was to evaluate the accuracy of the conventional imaging modalities (CT, MRI, US) and fine-needle aspiration cytology (FNAC) in the detection of lymph node metastases in the neck.

**Methods:**

Sixty two patients were included in the study, who underwent primary tumor resection and neck dissection. Preoperative nodal status was compared with postoperative histopathology nodal status. In our retrospective study, we reviewed the patient documentation. Statistical analysis of the data—with descriptive statistics and correlation analysis—was performed with Chi-square test.

**Results:**

The sensitivity of conventional imaging modalities and FNAC were 82.8% and 81.8%, respectively, while specificity were 73.9% and 100%, respectively. Positive predictive value calculated for imaging modalities and FNAC were 82.8%, 100%, respectively, while negative predictive values were 73.9% and 66.6%, respectively.

**Conclusion:**

Neither the sensitivity of imaging modalities (CT, MRI, US) nor FNAC reached 100%, none of these methods can definitively exclude the presence of regional tumor metastasis. According to these data, no permissive alteration should be allowed from the current guidelines (e.g. NCCN) based on imaging/FNAC examinations regarding the need for elective neck dissection.

## Purpose

The presence of cervical lymph node metastases is one of the most influential prognostic factors in head and neck squamous cell carcinomas. Adequate treatment based on precise preoperative diagnostic work-up is crucial for achieving the desired oncologic outcome [1]. The indication for neck dissection can be either therapeutic (for clinically N + necks) or elective (for clinically N0 necks). Most currently available guidelines recommend comprehensive neck dissection (levels I–V.) if preoperative examinations (physical examination, imaging, FNAC) reveal pathologic lymph nodes. Based on classic textbooks, elective treatment of the neck is recommended in clinically negative (cN0) necks when the probability of occult micrometastases exceeds 15–20%, which is embraced by the therapeutic guidelines, easing the everyday clinical decision making for certain tumor sites and stages [2].

Unilateral lymph node dissection is usually recommended, but bilateral dissection is warranted in tumors reaching the midline and due to the bilateral lymphatic drainage in tumors in certain locations (e.g. base of the tongue, soft palate, supraglottic larynx).

Management of the N0 neck is still a controversial area in the surgical treatment of head and neck tumors. It is known that the number of elective neck dissections in which the postoperative histopathological examinations reveal no metastatic lymph nodes is relatively high, however, the morbidity—such as hypesthesia, lymphedema, decreased head/neck, or arm mobility—of these procedures are also substantial in a varying degree, resulting in a decreased quality of life [3]. Moreover, there are cases when regional recurrence occurs over the years, adversely affecting overall survival. For this reason, careful preoperative investigation, diagnostic algorithms, and therapeutic recommendations are extremely important.

In our study, we aimed to evaluate the reliability of the preoperative investigations of our patients who underwent neck dissections by calculating the specificity, sensitivity, negative-, and positive predictive values regarding the presence/absence of metastatic lymph nodes.

## Methods

All patients enrolled in our study were diagnosed (histologically) and treated between September 2012 and August 2017 at the Department of Otolaryngology and Head-Neck Surgery, Semmelweis University, Hungary. The patients underwent upfront surgical treatment (resection of the primary and neck dissection) for cancers of the oral cavity, oropharynx, hypopharynx, or larynx.

Patients who were not diagnosed (by biopsy) at our tertiary referral center, had primaries in other head and neck sites (e.g. nasopharynx, nasal cavity, paranasal sinuses, salivary gland, or skin, furthermore those unknown primaries), or had previous head and neck oncological treatment were excluded from our study, thus, to maintain sample homogeneity, we had to exclude a large number of patients who underwent salvage surgery or were referred to our department from other hospitals.

The patients underwent head and neck imaging (CT or MRI) to determine the stage of their disease. All imaging studies were performed—according to the Hungarian regulations—at the regional imaging center indicated by the patient’s location (home addresses). Then, the patients were referred to the US-guided FNAC clinic of the 2nd Department of Pathology, Semmelweis University to obtain cytologic confirmation of any pathologic lymph node detected by ultrasound-expert cytologists.

In our retrospective study, we evaluated the patients’ medical records (discharge summaries, multidisciplinary tumor board (MTB) reports, histological reports, and imaging studies). The results of the preoperative examinations were correlated to the histopathological results of the neck dissection specimens. Both preoperative examination results (cTNM) and pathological TNM (pTNM) were evaluated according to the UICC 2010 version 7 TNM classification [4]. When reviewing the radiological findings, lymph nodes were considered abnormal if they had previously been considered abnormal by the radiologist if they were larger than 10 mm in their shortest diameter, contained central necrosis, or had a round shape [5].

Bilateral neck dissections were performed in 12 cases where the preoperative FNAC results were the same for the two sides of the neck. However, in two cases, discordant (one side positive, the other side negative) FNAC results were obtained, while bilateral metastases were reported in the pathological report of the neck dissection specimen. Both cases were considered false negatives in our study.

Both FNAC and imaging were done as part of the clinical routine, so they were performed by various physicians (radiologists and cytologists).

## Statistical procedure

The statistical analysis of the data—with descriptive statistics and correlation analysis—was performed with the Chi-square test (Fisher’s exact test) using SPSS Statistics for Mac v.22.0 (IBM Corp., Armonk, NY).

## Results

We identified 62 patients who met the criteria described above. In terms of their gender distribution, we examined 54 men and 8 women. The mean age of the patients at the time of diagnosis was 61 years (43–77 years). Regarding the localization of the primaries, most patients had hypopharyngeal tumors (32.3%), followed (in descending order of frequency) by supraglottic (22.6%), transglottic (21%), glottic (9.7%) laryngeal cancer, oropharyngeal (9.7%), oral (3.2%), and subglottic laryngeal (1.6%) tumors, respectively (Table 1).

**Table 1:** Distribution of tumors by localization

Localization	%
Oral	3.2
Oropharynx	9.7
Hypopharynx	32.3
Supraglottic	22.6
Glottic	9.7
Transglottic	21
Subglottic	1.6

Regarding preoperative stages, T1 (T1a and T1b for glottic cancers) stage tumors were the least common, only 4.8%, while T2, T3 stage tumors accounted for 32.3% − 32.3%, slightly less than T4a and T4b stages: 30.6%.

In terms of preoperative N stage of the tumors, 53.2% were N0, 16.1% were N1, 16.1% were N2a, 8.1% were N2b, 4.8% were N2c, and 1.6% of them were stage N3, so 53.2% of the patients received elective neck dissection and 46.7% received therapeutic, comprehensive neck dissection.

The most common therapies recommended by MTB were total laryngectomy with ipsilateral neck dissection (ND) (38.7%), partial laryngectomy with ipsilateral ND (27.4%), and total laryngectomy with bilateral NDs (14.5%). The procedures performed are listed in Table 2.

**Table 2:** Types of surgeries performed

Therapy	%
Partial laryngectomy with ipsilateral neck dissection	27.4
Total laryngectomy with ipsilateral neck dissection	38.7
Pharyngotomy with ipsilateral neck dissection	8.1
Transoral resection with ipsilateral neck dissection	6.5
Partial laryngectomy bilateral neck dissections	4.8
Total laryngectomy bilateral neck dissections	14.5

Concerning the distribution of imaging techniques used for head and neck cancer staging, the majority of patients underwent CT scan (84%), 10% had MRI scan, and 3.2% only had ultrasound scan.

As anticipated, we found a significant (*p* < 0.001) correlation between the nodal stage described by imaging (CT, MRI, US) and the histopathology report (pN1, pN2a, pN2b, pN2c, pN3 summarized as pN + and N0 as pN −). However, the discordance was noteworthy: 73.9% of the radiologic N0 cases were truly negative, while 26.1% had false negative preoperative imaging results in terms of the nodal stage (Tables 3, 4, 5, 6).

**Table 3:** Relationship between CT report and pathological lymph node status

% (No)	Pathology lymph node status (pN)	
Lymph node status described on the CT	Negative	Positive
Negative	72.2 (13)	27.8 (5)
Positive	15.6 (5)	84.4 (27)

**Table 4:** Relationship between MRI report and pathological lymph node status

% (No)	Pathology lymph node status (pN)	
Lymph node status described on the MRI	Negative	Positive
Negative	80 (4)	20 (1)
Positive	0	100 (1)

**Table 5:** Relationship between US report and pathological lymph node status

% (No)	Pathology lymph node status (pN)	
Lymph node status described on the US	Negative	Positive
Negative	0 (0)	0 (0)
Positive	50 (1)	50 (1)

**Table 6:** Relationship between imaging study report and pathological lymph node status

% (No)	Pathology lymph node status (pN)	
Lymph node status described on the imaging techniques combined	Negative	Positive
Negative	73.9 (17)	26.1 (6)
Positive	17.1 (6)	82.9 (29)

Although there was a significant association between FNAC results and histopathologic lymph node status (*p* < 0.001), the rate of false-negative cases was surprisingly high: only 66.7% of neck statuses considered negative by FNAC were proven to be negative by the histopathologic examinations of the surgical specimens. As expected, 100% of the lymph nodes diagnosed positive by the FNAC were also positive histopathologically (Table 7).

**Table 7:** Relationship between FNAC results and pathological lymph node status

% (No)	Pathology lymph node status (pN)	
FNAC	Negative	Positive
Negative	66.7 (12)	33.3 (6)
Positive	0 (0)	100 (27)

Based on the above, the sensitivity of imaging modalities was calculated to be 82.8%, while specificity was 73.9%, the positive predictive value was 82.8%, and the negative predictive value was 73.9%. On the other hand, regarding the FNAC, the sensitivity, specificity, negative and positive (Table 8).

**Table 8:** Percentage values for CT separately, imaging techniques combined, and FNAC examinations

%	CT*	Imaging techniques combined	FNAC
Sensitivity	84.4	82.8	81.8
Specificity	72.2	73.9	100
Positive predictive value	84.4	82.8	100
Negative predictive value	72.2	73.9	66.6

*For MRI and US, specificity and sensitivity could not be calculated separately because of low number of participants

The clinical TNM stage (along with clinical lymph node status—cN) is finally established by the multidisciplinary tumor board based on physical examination, imaging, and FNAC results. In our study, 96.6% of clinically N + cases were proven to be pathologically positive. However, only 69.7% of cases preoperatively evaluated as N0 had pN0 stage based on the histopathological examination of the dissection specimen, accordingly, 30.3%. of cases with cN0 stages were false negatives.

## Discussion

Precise staging workup is crucial before decision making regarding the treatment of head and neck cancer patients. In our work, we aimed to determine the diagnostic accuracy of preoperative imaging modalities (CT, MRI, US) and FNAC in terms of clinical and pathological nodal stages. Previous reports found different diagnostic reliability of certain diagnostic modalities.

Knappe and colleagues compared the US-guided fine-needle aspiration cytology results with the histopathology samples of the patients undergone neck dissections. They found FNAC sensitivity to be 89.2% and specificity to be 98.1% [6].

According to Takes et al., the US-guided FNAC had 77% sensitivity and 100% specificity in the detection of cervical lymph node metastases. This research highlights that the method has low inter-observer variability—and therefore its widespread use is encouraged [7].

Dammann et al. evaluated the efficiency of CT, MRI, and FDG-PET in the preoperative staging of SCC of the head and neck region. They found CT sensitivity to be 80%, specificity to be 93%, while MRI sensitivity was 93%, specificity was 95%. In the case of ambivalent cases, the study recommended PET for an additional diagnostic procedure [8].

Adams et al. compared the accuracy of conventional imaging studies (US, CT, MRI) with FDG-PET. CT showed 82% sensitivity and 85% specificity, while MRI had 80% sensitivity and 79% specificity. The ultrasound showed a lower sensitivity with 72%. FDG-PET and conventional imaging showed a statistically significant correlation in the detection of cervical lymph node metastases (PET vs. CT, *p* = 0.017; PET vs. MRI, *p* = 0.012; PET vs. US, *p* = 0.0001) [9].

W. van den Brekel et al. performed a study evaluating the value of US and US-guided FNAC for the assessment of N0 neck status. US detected the occult lymph node metastasis with 60% sensitivity and 77% specificity, while the US-guided FNAC showed 76% sensitivity and 100% specificity. Due to this high accuracy, this work considered US-guided FNAC as an important technique in the N0 neck examination [10].

De Bondt and colleagues performed a meta-analysis comparing US, US-guided FNAC, CT, and MRI in the detection of lymph node metastases in head and neck cancer patients. There was a large variability not only in the accuracy of the techniques but also in the cut-off levels in the size and morphology of the lymph nodes considered as positive. US-guided FNAC showed the highest diagnostic odds ratios (DOR = 260) compared to US (DOR = 40), CT (DOR = 14), MRI (DOR = 7). US showed the highest sensitivity with 87%, while the highest specificity was linked to US-guided FNAC (98%). The meta-analysis concluded that the US-guided FNAC showed the best diagnostic performance, while the US, CT, and MRI were less effective in this regard [11].

In Sumi et al.’s research, CT and MRI were compared by examining changes in lymph node parenchyma (cancer nest, necrosis, and keratinization). Small-sized (shorter axial diameter less than 10 mm) and large-sized (shorter axial diameter 10 mm or larger) lymph nodes were evaluated separately. In the detection of small metastatic lymph nodes, MRI performed significantly better, sensitivity, specificity, positive predictive value, and negative predictive value were 83%, 89%, 89%, and 84%, respectively, while those for CT-scan were 68%, 79%, 79%, and 72%, respectively. There was no significant difference when examining large lymph nodes between the two imaging techniques (MRI: sensitivity 100%, specificity 98%, positive predictive value 99%, negative predictive value 100%, CT: sensitivity 98%, specificity 89% positive predictive value 95%, negative predictive value 97%) [12].

In Akoglu et al.’s paper, the diagnostic efficacy of conventional imaging (US, CT, MRI) in the detection of cervical lymph node metastasis was compared. The following results were obtained in their research: CT sensitivity 77.7%, specificity 85.7%, positive predictive value 91.3%, negative predictive value: 66.6%, MRI sensitivity 59.2%, specificity 92.8%, positive predictive value 94.1%, negative predictive value: 54.1%, US sensitivity 81.4%, specificity 64.2%, positive predictive value 81.4%, negative predictive value: 64.2%. US and CT performed better than MRI, however, there was no significant difference between them. They also concluded that because of the low negative predictive value, neither method could be used to accurately detect cervical metastases [13].

In their retrospective research, Yoon et al. investigated how effectively CT, MRI, US and FDG-PET-CT, and a combination of these modalities detect cervical lymph node metastasis. As a result, they had a sensitivity of 77% and a specificity of 99.4% for CT and MRI, sensitivity of 78.4%, and specificity of 98.5% for US. In conclusion, the results of the study showed that sensitivities and specificities of CT, MR, US, and PET/CT appeared to be similar in the detection of cervical lymph node metastases. The use of the four techniques combination yielded improved sensitivity compared with the single use of these techniques, but without a statistically significant difference [14].

Interpreting our results, we can see that neither the sensitivity of the imaging methods (CT, MRI, US) nor the sensitivity of fine-needle aspiration reached 100%. Also, the rate of false-negative results was relatively high for both methods: 26.1% for imaging techniques and 33.3% for FNAC. Thus neither method can definitively establish the presence of cervical metastasis. This is especially important in T1, T2 stage tumors because the N0 cervical stage is the most common in these cancers.

Our results do not differ substantially from those previously published, underlining that no current diagnostic method is reliable enough to exclude the presence of metastatic cervical lymph nodes (Table 9).

**Table 9:** Sensitivity and specificity of FNAC, CT, MRI expressed in percentage

Authors	Type of the study	FNAC		CT		MRI	
		Sens	Spec	Sens	Spec	Sens	Spec
Knappe et al.	Prospective, single institution	89.2	98.1				
Takes et al.	Prospective, multi-institute	77	100				
Dammann et al.	Prospective, single institution			80	93	93	95
Adams et al.	Prospective, single institution			82	85	80	79
Brekel et al.	Prospective, single institution	76	100				
De Bondt et al.	Meta-analysis	80	98	81	76	81	63
Sumi et al.	Retrospective,single institution			68	79	83	89
Akoglu et al.	Prospective, single institution			77.7	85.7	59.2	92.8
Yoon et al.	Retrospective, multi-institute			77	99.4	77	99.4
Our research	Retrospective, single institution	81.8	100	82.8	73.9		

## Conclusion

We cannot decide not to perform an elective neck dissection based on the ’negative’ result of the preoperative staging workup, current therapeutic guidelines (e.g. NCCN) should be followed in therapeutic decision-making.
